# Blood count derangements after sepsis and association with post-hospital outcomes

**DOI:** 10.3389/fimmu.2023.1133351

**Published:** 2023-02-28

**Authors:** Scott J. Denstaedt, Jennifer Cano, Xiao Qing Wang, John P. Donnelly, Sarah Seelye, Hallie C. Prescott

**Affiliations:** ^1^ Division of Pulmonary and Critical Care Medicine, Department of Internal Medicine, University of Michigan Medical School, Ann Arbor, MI, United States; ^2^ VA Center for Clinical Management Research, Ann Arbor, MI, United States; ^3^ Department of Learning Health Sciences, University of Michigan, Ann Arbor, MI, United States

**Keywords:** sepsis, survivor, anemia, neutrophil lymphocyte ratio (NLR), complete blood cell (CBC) count

## Abstract

**Rationale:**

Predicting long-term outcomes in sepsis survivors remains a difficult task. Persistent inflammation post-sepsis is associated with increased risk for rehospitalization and death. As surrogate markers of inflammation, complete blood count parameters measured at hospital discharge may have prognostic value for sepsis survivors.

**Objective:**

To determine the incremental value of complete blood count parameters over clinical characteristics for predicting 90-day outcomes in sepsis survivors.

**Methods:**

Electronic health record data was used to identify sepsis hospitalizations at United States Veterans Affairs hospitals with live discharge and relevant laboratory data (2013 to 2018). We measured the association of eight complete blood count parameters with 90-day outcomes (mortality, rehospitalization, cause-specific rehospitalizations) using multivariable logistic regression models.

**Measurements and main results:**

We identified 155,988 eligible hospitalizations for sepsis. Anemia (93.6%, N=142,162) and lymphopenia (28.1%, N=29,365) were the most common blood count abnormalities at discharge. In multivariable models, all parameters were associated with the primary outcome of 90-day mortality or rehospitalization and improved model discrimination above clinical characteristics alone (likelihood ratio test, p<0.02 for all). A model including all eight parameters significantly improved discrimination (AUROC, 0.6929 v. 0.6756) and reduced calibration error for the primary outcome. Hemoglobin had the greatest prognostic separation with a 1.5 fold increased incidence of the primary outcome in the lowest quintile (7.2-8.9 g/dL) versus highest quintile (12.70-15.80 g/dL). Hemoglobin and neutrophil lymphocyte ratio provided the most added value in predicting the primary outcome and 90-day mortality alone, respectively. Absolute lymphocyte count added little value in predicting 90-day outcomes.

**Conclusions:**

The incorporation of discharge complete blood count parameters into prognostic scoring systems could improve prediction of 90-day outcomes. Hemoglobin had the greatest prognostic value for the primary composite outcome of 90-day rehospitalization or mortality. Absolute lymphocyte count provided little added value in multivariable model comparisons, including for infection- or sepsis-related rehospitalization.

## Introduction

An estimated 38 million patients survive hospitalization for sepsis each year (1), many of whom are re-hospitalized or die within 90 days of discharge (2, 3). While there has been increased attention to longer-term morbidity and mortality after sepsis, targeted therapies to prevent these sequelae are lacking (4–6). In addition, predicting post-acute sequelae, particularly rehospitalization, remains challenging with predictive models performing only modestly (7–10). Heterogeneity in sepsis and sepsis survivorship is a major factor complicating the development of predictive models and application of therapies directed at improving long-term outcomes (4, 11).

Persistent inflammation is a source of biological heterogeneity in sepsis survivors that is independently associated with increased risk for long-term rehospitalization and death (12). It is hypothesized to contribute to poor outcomes particularly through enhancing the risk of secondary infections leading to rehospitalization (13–15). Therefore, identifying patients with persistent inflammation at hospital discharge may improve prediction of adverse events and help guide targeted therapies. Prior studies assessing risk factors for post-sepsis morbidity have largely focused on non-modifiable factors such as age, comorbidities, and pre-hospitalization healthcare utilization (2, 8, 16). Biological variables, including measures of persistent inflammation at hospital discharge, have been infrequently evaluated in sepsis survivors and are not currently incorporated into predictive models (7, 8, 12). A hallmark of the acute inflammatory response of sepsis is dramatic changes in complete blood count and leukocyte differential (CBCD) parameters as a result of altered production, consumption, and death of blood cells (17, 18). The magnitude and duration of deviations from normal physiologic range are associated with in-hospital mortality and hospital-acquired infections (19–25). In addition to leukocyte parameters, alterations of hemoglobin and platelet count are also associated with dysregulated inflammation and acute sepsis mortality (21, 26). As surrogate markers of the inflammatory response, we hypothesized that alterations of CBCD parameters measured at the time of hospital discharge may be associated with risk for post-acute sequalae and could improve predictive models.

In this study, we examined the association of CBCD parameters at hospital discharge with post-hospital outcomes. To do this, we evaluated a national cohort of sepsis survivors treated in the Veterans Affairs (VA) healthcare system, for whom both detailed electronic health record data and post-hospital outcomes were available (27). We examined the association of eight CBCD parameters with 90-day all-cause rehospitalization or mortality, as well component outcomes and cause-specific rehospitalizations (infection-related, sepsis-related). Finally, we quantified the additive value of CBCD parameters in predicting 90-day outcome as compared to clinical data alone using multivariable logistic regression models.

## Methods

### Study setting

The U.S. Veterans Affairs (VA) healthcare system is an integrated healthcare system that provides comprehensive medical care to over 6 million veterans (28). During the study period, the VA used a single electronic health record (EHR) system, archived in a central repository (the Corporate Data Warehouse, CDW) accessible for research (28). This study was performed under waiver of informed consent and was approved by Ann Arbor VA Institutional Review Board under the study name “ Understanding and Informing Early Hospital Antibiotic Prescribing for Potential Infection in the VA”, protocol number 1597404 and was approved on 10/31/2019. All procedures were followed in accordance with the ethical standards of the responsible committee on human experimentation and with the Helsinki Declaration of 1975.

### Study cohort

We identified sepsis hospitalizations with live discharge and relevant laboratory data from 138 nationwide VA hospitals (2013 to 2018). Sepsis hospitalizations were identified as in prior work (27, 29–31) using EHR clinical data, akin to the approach used in the Centers for Disease Control and Prevention’s (CDC) adult sepsis event surveillance definition (cdc.gov, CDC Hospital Toolkit). Specifically, we identified hospitalizations admitted through the emergency department (ED) with evidence of 1) suspected infection and 2) acute organ dysfunction (27). Suspected infection was defined as 2+ systemic inflammatory response syndrome (SIRS) criteria (32), initiation of systemic antimicrobials (oral or intravenous) within 48 hours of ED arrival, and continuation of antimicrobials for at least 4 days (31), acute organ dysfunction was defined as ≥ 1 acute organ dysfunction present within 48 hours of emergency department arrival: acute renal dysfunction, acute liver dysfunction, acute hematologic dysfunction, lactate > 2.0 mmol/L, receipt of invasive mechanical ventilation, and receipt of systemic vasopressors. Acute renal, liver, and hematologic dysfunction were defined based on worsening of creatinine, total bilirubin, and platelet count respectively from baseline, where baseline was defined as the best laboratory value during hospitalization or the preceding 6 months (27). We defined live discharge as being alive on the calendar day of discharge and the day following (to exclude patients discharged home at end of life). We defined relevant laboratory data as having at least one laboratory of interest white blood cell count (WBC), hemoglobin (Hgb), or platelet count (Plt) on the calendar day of discharge or day prior. Patient and hospitalization data, including demographics, comorbidities (**Supplemental Table 1**), and hospital treatments were extracted from the VA Corporate Data Warehouse (CDW), as described previously (29, 30).

### Exposure: Discharge CBCD laboratories

For each hospitalization, we extracted laboratory values during hospitalization if drawn on either the calendar day of discharge or day prior, including: total WBC, Hgb, Plt, absolute neutrophil count (ANC), and absolute lymphocyte count (ALC). Laboratory values were cleaned and standardized using previously described methods, with publicly-available statistical code (https://github.com/CCMRcodes/CBC_Parameters-) (30). After exclusion of non-physiologic laboratory values (**Supplemental Table 2**), the top and bottom 1% of laboratories were trimmed (**Supplemental Table 3**), reasoning that these extreme values—while physiologically-plausible—were consistent with hematologic malignancy or bone marrow failure, and therefore known to be associated with poor outcomes. After exclusion of the top and bottom 1%, we calculated three combination variables from contemporaneous component values: (1) neutrophil lymphocyte ratio (NLR = ANC/ALC) (33); (2) platelet lymphocyte ratio (PLR = platelet count/ALC) (34); and (3) systemic immune-inflammation index (SII = (ANC x platelet count)/ALC) (35).

### Outcomes

The primary outcome was rehospitalization or death within 90 days of index sepsis hospitalization discharge. Secondary outcomes were 90-day mortality, 90-day rehospitalization, 90-day re-hospitalization for potential infection, and 90-day rehospitalization for sepsis. Rehospitalization for potential infection was defined as readmission through the emergency department with ≥2 SIRS criteria and treatment with antimicrobials within 48 hours of arrival (27). Rehospitalization for sepsis additionally required continuing antimicrobials for ≥4 calendar days (or death prior to 4 days while on consecutive days of therapy) and acute organ dysfunction, as for index sepsis hospitalizations.

### Statistical analysis

To evaluate the association of each CBCD parameter with the primary outcome, Cox proportional hazards models were fit and CBCD parameters were modeled flexibly using restricted cubic splines. We also fit models with CBCD parameters classified by quintiles and reported Kaplan-Meier failure curves. We used Royston-Sauerbrei D-statistics (R_D_^2^) to quantify the separation of failure curves by CBCD parameter (36). To understand the incremental predictive value of discharge CBCD parameters, we fit multivariable logistic regression models and used the area under the receiver operating characteristics (AUROC) and likelihood ratio tests to determine significant improvement over a base model using clinical variables alone. Clinical variables selected for inclusion in the base model were all previously associated with risk of post-hospitalization death or rehospitalization in other publications (7–9, 37). Additionally, we fit a full model using clinical variables and all eight CBCD parameters. For this full model, we assessed calibration visually and assessed for improvement in maximum and mean calibration error compared to the base clinical model (38, 39). Data management and analysis were performed using SQL, SAS (SAS Institute Inc.), and R (R Foundation for Statistical Computing, https://www.R-project.org/). All statistical code for the predictive models is available online (https://github.com/CCMRcodes/CBC_Parameters-).

## Results

### Study cohort

**Figure 1:**
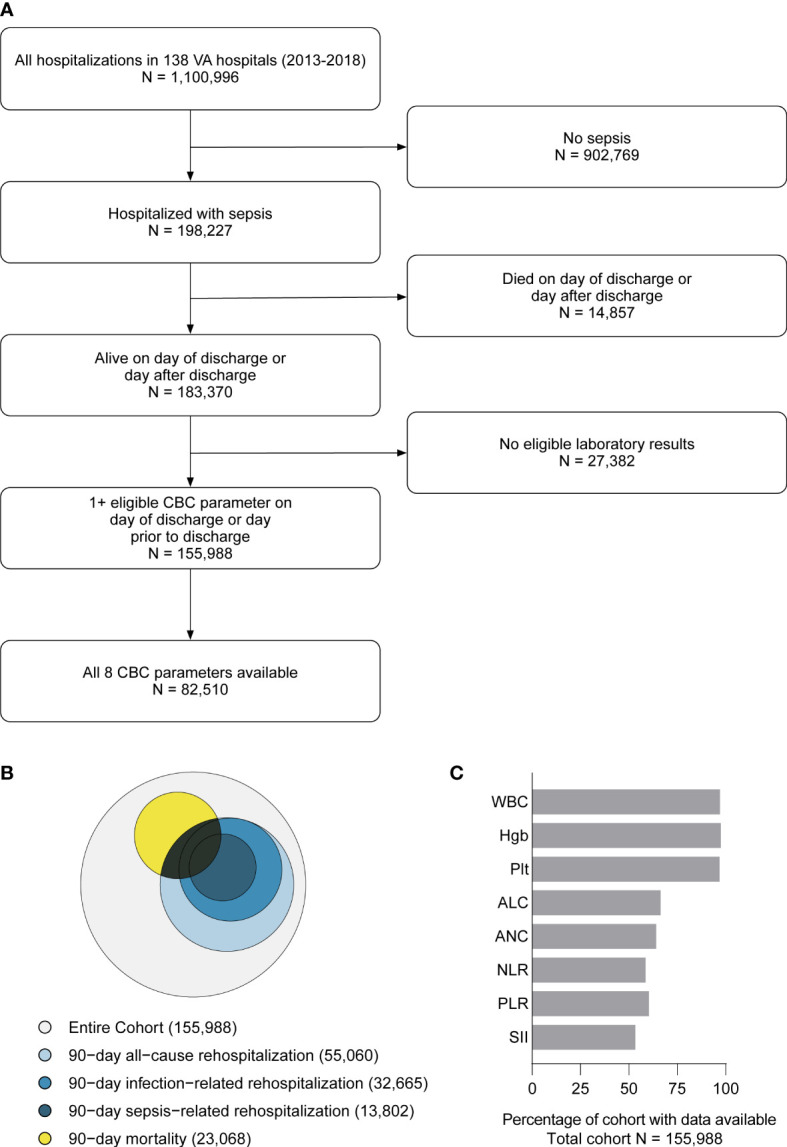
Flow diagram showing study inclusion/exclusion criteria and the final cohort **(A)**. Euler diagram (proportional Venn diagram) showing the relative proportion of the cohort experiencing each component 90-day outcome, including mortality (yellow), all-cause re-hospitalization (light blue), infection-related re-hospitalization (blue), and sepsis-related rehospitalization (dark blue) **(B)**. Each discharge CBCD parameter was available for the majority of the hospitalizations **(C)**. WBC, total white blood cell count. Hgb, hemoglobin. Plt, platelet count. ANC, absolute neutrophil count. ALC, absolute lymphocyte count. NLR, neutrophil-to-lymphocyte ratio (NLR = ANC/ALC). PLR, platelet-to-lymphocyte ratio (PLR = Plt/ALC). SII, systemic immune-inflammation index (SII = ANC x PLR/ALC).

During the study period (2013-2018), there were 1,100,966 SIRS-positive admissions *via* the emergency department to 138 VA hospitals. 198,227 (18.0%) hospitalizations met surveillance criteria for sepsis, of which 183,370 (92.5%) were discharged alive, and 155,988 (85.1% of live discharges) had eligible laboratory data near discharge and were included in the study (**Figure 1A**). 69,062 (44.3%) experienced the primary composite outcome of 90-day mortality or rehospitalization (**Figure 1B**), including 23,068 (14.8%) with 90-day mortality, 55,060 (35.3%) with all-cause rehospitalization, 32,665 (20.9%) with infection-related rehospitalization, and 13,802 (8.9%) with sepsis-related rehospitalization. CBCD parameters near discharge were available for a majority of hospitalizations, including WBC (97.1%), Hgb (98.1%), Plt (97.5%), ANC (64.6%), ALC (66.9%), NLR (59.2%), PLR (60.9%), and SII (54.0%) (**Figure 1C**). The cohort was a median 68 years (IQR: 54, 82), 96.3% male, and 71.4% White (**Table 1**). Hospital length of stay was a median of 6 (IQR: 4, 10) days, and 46,061 (29.5%) were treated in an ICU. Vasopressor treatment was used in 11,827 (7.6%) while the most common acute organ dysfunctions were acute kidney injury (61.1%), elevated lactate (43.9%), and acute hematologic dysfunction (13.1%) (**Supplemental Table 4**).

**Table 1:** Characteristics of Sepsis Cohort, stratified by composite outcome.

Patient Characteristics	Total cohort (n = 155,988)	No mortality or rehospitalization (n = 86926)	90-day all-cause mortality or rehospitalization (n = 69062)
**Age, median (IQR)**	68 (62-76)	68 (61-75)	69 (63-77)
**Sex, M, %**	96.3	96.0	96.8
Race, %			
Black	20.9	20.6	21.2
White	71.4	71.4	71.3
Other	2.2	2.3	2.0
Unknown	5.4	5.5	5.3
**Comorbidity count, median (IQR)**	6 (4-8)	5 (3-7)	7 (5-9)
**Acute organ dysfunction count, median (IQR)**	1 (1-2)	1 (1-2)	1 (1-2)
**ICU admission, n (%)**	46061 (29.5)	23274 (26.8)	22787 (33.0)
**Any surgery during hospitalization**	20,996 (13.5)	12,112 (13.9)	8884 (12.9)
**Hospital LOS, median (IQR)**	6 (4-10)	6 (4-9)	7 (5-11)
Discharge blood parameter, median (IQR)			
WBC (10^3^/µL)	8.39 (6.20-10.90)	8.31 (6.30-10.80)	8.40 (6.09-11.10)
Hemoglobin (g/dL)	10.7 (9.2-12.20)	11.2 (9.70-12.6)	10.1 (8.80-11.7)
Platelet count (10^3^/µL)	214 (152-292)	219 (160-294)	208 (139-289)
ANC (10^3^/µL)	5.65 (3.80-7.90)	5.6 (3.83-7.71)	5.72 (3.80-8.10)
ALC (10^3^/µL)	1.3 (0.90-1.85)	1.4 (1.00-1.90)	1.2 (0.80-1.73)
NLR	4.11 (2.59-6.64)	3.86 (2.48-6.12)	4.48 (2.77-7.40)
PLR	161 (109-238)	156 (108-227)	167.59 (111-252)
SII	888 (490-1560)	851(484-1462)	948.23 (501-1701)

Comorbidity count determined using 30 Elixhauser comorbidities.

WBC, total white blood cell count. ANC, absolute neutrophil count. ALC, absolute lymphocyte count. NLR, neutrophil-to-lymphocyte ratio (NLR = ANC/ALC). PLR, platelet-to-lymphocyte ratio (PLR = Plt/ALC). SII, systemic immune-inflammation index (SII = ANC x PLR/ALC).

### CBCD parameters

The median (IQR) for each parameter was WBC 8.39 (6.20-10.90) x 10^3^/µl; Hgb 10.7 (9.2-12.20) g/dl; Plt 214 (152-292) x 10^3^/µl; ANC 5.65 (3.80-7.90) x 10^3^/µl; ALC 1.3 (0.90-1.85) x 10^3^/µl; NLR 4.11 (2.59-6.64); PLR 161 (109-238); and SII 888 (490-1560). Nearly all patients had at least one CBCD parameter outside normal range (**Supplemental Table 5**), most commonly for hemoglobin with 143,162 (93.6%) of measurements occurring below 14 g/dl. The frequencies of other parameters below the lower-limit of normal range were ALC 29,365 (28.1%), Plt 36,817 (24.2%), WBC 11,566 (7.6%), and ANC 3,586 (3.6%). The frequencies of parameters occurring above the upper-limit of normal range were WBC 36,534 (24.0%), ANC 24,702 (23.9%), Plt 12,175 (8.0%), and ALC 685 (0.7%).

### Association of CBCD parameters with 90-day mortality or rehospitalization among sepsis survivors

Patients experiencing the primary composite outcome were older (69 (63-77) v. 68 (61-75), p<0.001), had more comorbidities (7 (5-9) v. 5 (3-7), p<0.001), and experienced a higher burden of acute organ dysfunction during sepsis hospitalization (renal 63.6% v. 59.2%, hematologic 16.7% v. 10.3%, hepatic 13.72% v. 12.33%, circulatory 8.18% v. 7.11%, all p<0.001) (see **Table 1** and **Supplemental Table 4**). They were more commonly treated in an ICU (33.0% v. 26.8%, p<0.001) and had longer lengths of hospitalization (7 (5-11) v. 6 (4-9) days, p<0.001). Surgery during hospitalization was less common in patients with the primary outcome as compared to those without (12.9% v. 13.9%, p<0.001). There was no difference in median (IQR) WBC count in patients with the primary outcome as compared to those without. However, median Hgb, Plt, and ALC were all lower, while ANC, NLR, PLR, and SII were all higher among patients who experienced the primary outcome. Patient characteristics were similar for secondary outcomes of 90-day mortality, all-cause rehospitalization, and cause-specific rehospitalization (**Supplemental Tables 6, 7**).

**Figure 2:**
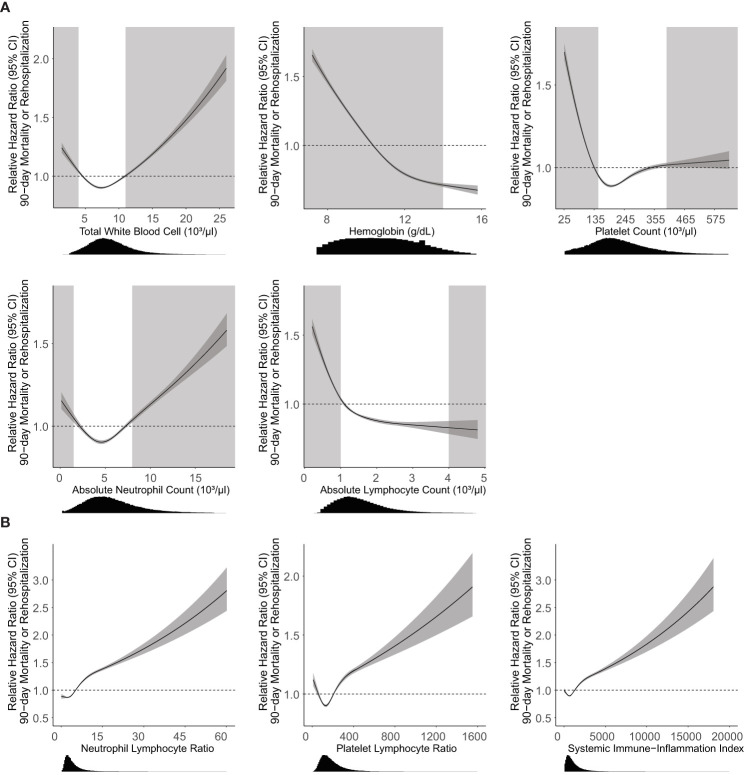
Association of CBCD parameters with 90-day mortality or rehospitalization. Unadjusted Cox proportional hazard regression (95% CI, dark gray) with relative hazard ratio shown for individual **(A)** and combination **(B)** CBCD parameters. Histogram distributions of parameters shown below the x-axis. Light gray boxes indicate CBCD parameters outside of the normal clinical range for individual parameters. There are no well-established normal ranges for NLR, PLR, or SII for the general population. Neutrophil Lymphocyte Ratio = absolute neutrophil count/absolute lymphocyte count. Platelet Lymphocyte Ratio = platelet count/absolute lymphocyte count. Systemic Immune-Inflammation index = (absolute neutrophil count x platelet count)/absolute lymphocyte count.

In bivariate analysis, all CBCD parameters had a strong association with the primary outcome (**Figure 2A**). Relative hazard of the outcomes increased for higher WBC and ANC, and lower ALC, Hgb, and Plt. Deviations in each parameter beyond the established normal range (**Figure 2**, light gray boxes) were associated with the greatest increases in hazard of the primary outcome for all parameters, except for Plt and ALC which had relatively flat relationships with the hazard of primary outcome above the upper-limit of normal. Combined CBCD parameters showed similar relationships with the primary outcome (**Figure 2B**).

### Quantifying the prognostic value of CBCD parameters

**Figure 3:**
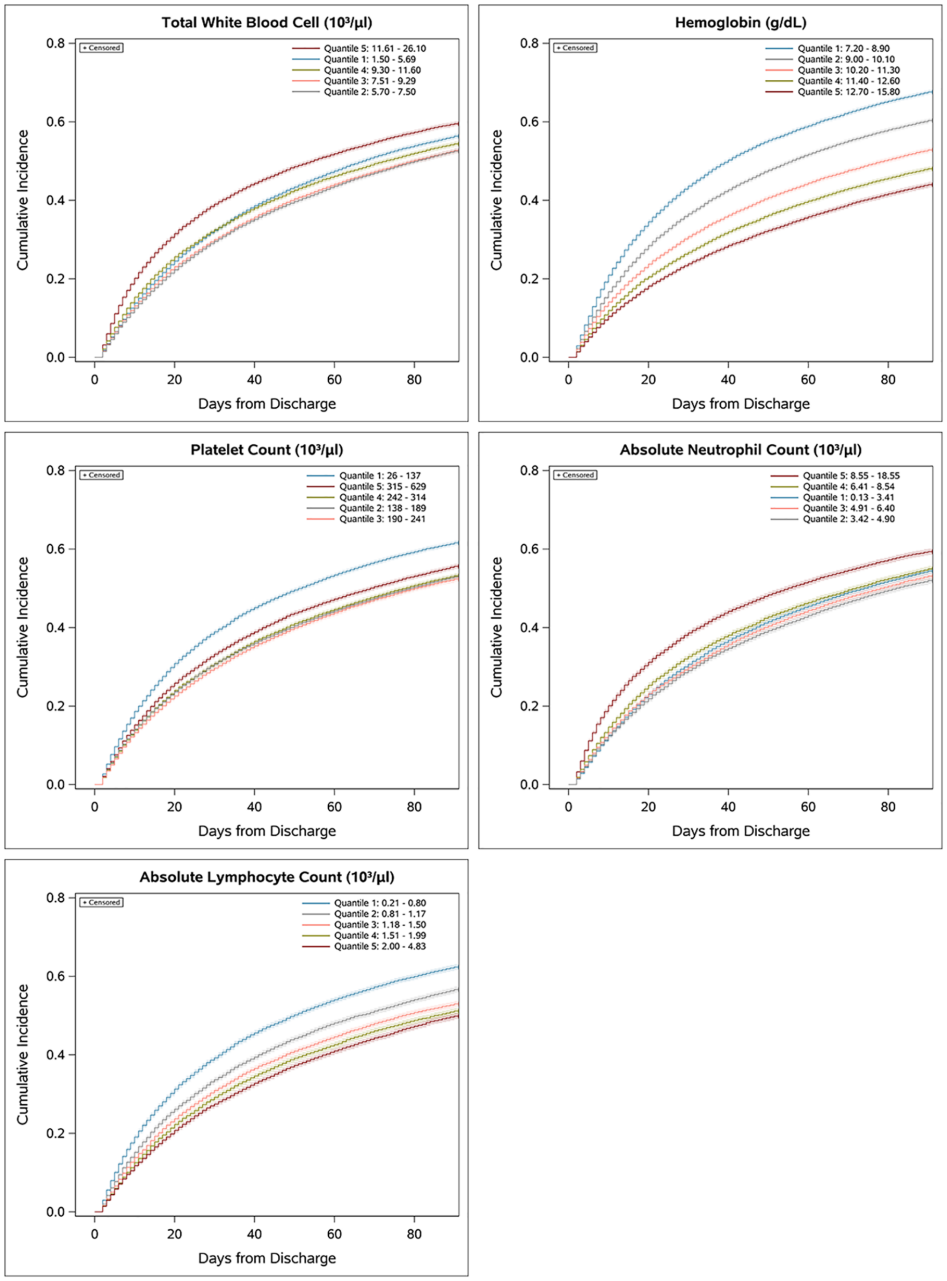
Association of individual CBCD parameters and cumulative incidence of 90-day mortality or rehospitalization. Univariate Kaplan-Meier failure plots showing cumulative incidence of the primary outcome by quintile.

To determine the relative predictive value of individual CBCD parameters, we first evaluated the association of CBCD parameters with the cumulative incidence of the primary outcome over time using Kaplan-Meier failure plots by quintile of individual (**Figure 3**) and combined (**Supplemental Figure 1**) CBCD parameters. Hgb had the greatest separation in outcomes by quintile, with a nearly 1.5 fold increased incidence of the primary outcome in the lowest quintile (7.2-8.9 g/dL) versus highest quintile (12.70-15.80 g/dL). To quantify the prognostic separation of Kaplan-Meier failure estimates and the proportion of variation explained by each parameter across all outcomes, the Royston-Sauerbrei R^2^_D_ statistic was calculated (**Table 2**). For the primary outcome of 90-day mortality or rehospitalization, the highest R^2^_D_ values were observed for Hgb (0.039), NLR (0.015), and ALC (0.012). For 90-day mortality alone, more substantial increases in prognostic separation were observed among Hgb (0.121) and NLR (0.117). Hgb also had the most prognostic separation for 90-day all-cause (0.267), infection-related (0.025), and sepsis-related (0.067) rehospitalization.

**Table 2:** Prognostic separation (R^2^_D_) of Kaplan-Meier failure plots CBCD parameters by outcome.

CBC Parameter	90-day mortality or rehospitalization	90-day mortality	90-day rehospitalization (all cause)	90-day rehospitalization (potential infection)	90-day rehospitalization (sepsis)
**WBC**	0.005	0.031	0.001	0.006	0.008
**Hemoglobin**	0.039	0.121	0.267	0.025	0.067
**Platelet count**	0.006	0.028	0.004	0.006	0.016
**ANC**	0.005	0.040	0.004	0.003	0.003
**ALC**	0.012	0.080	0.004	0.006	0.008
**NLR**	0.015	0.117	0.002	0.005	0.005
**PLR**	0.005	0.027	0.002	0.002	0.003
**SII**	0.008	0.054	0.002	0.003	0.004

R^2^_D_ - Royston-Sauerbrei D-statistic: estimates the separation between survival distributions allowing for comparison of relative prognostic value among multiple parameters and outcomes

WBC, total white blood cell count; ANC, absolute neutrophil count; ALC, absolute lymphocyte count; NLR, neutrophil-to-lymphocyte ratio (NLR = ANC/ALC); PLR, platelet-to-lymphocyte ratio (PLR = Plt/ALC); SII, systemic immune-inflammation index (SII = ANC x PLR/ALC).

### Additional predictive value of CBC parameters to clinical characteristic in prediction of 90-day outcome

Patient demographics and severity of index sepsis hospitalization are known to be associated with risk of mortality and rehospitalization in sepsis survivors (2, 8). We sought to determine the incremental predictive value of CBCD parameters for 90-day outcomes beyond these clinical characteristics by fitting sequential multivariable logistic models and comparing their AUROC (**Table 3**). To allow for direct comparisons across models, this analysis was restricted to 82,510 (53%) hospitalizations with all eight CBCD parameters available at discharge (differences in the characteristics of included *vs.* excluded sepsis hospitalizations were negligible, as shown in **Supplemental Table 8**). We found that clinical charactersitics alone had fair discrimination in predicting 90-day mortlity or rehospitalization (AUROC, 0.6756). There was better discrimination for 90-day mortality alone (AUROC, 0.7590), but worse discrimination for 90-day all-cause rehospitalization (AUROC, 0.6318) and cause-specific rehospitalization for potential infection (AUROC, 0.6289) or sepsis (AUROC, 0.6381). A full model including clinical characteristics and all eight CBCD parameters significantly improved discrimination for all outcomes and improved calibration (**Supplemental Figure 2**). Relative to the base model, the full model improved predicted risk across all risk deciles for the primary outcome with a 50% reduction in mean calibration error and appropriate reclassification of 1% of the hospitalizations (n = 753 appropriately reclassified out of 82,510 total hospitalizations) (**Supplemental Tables 9, 10**). Individual CBCD parameters provided marginal improvements in discrimination of the primary outcome, with Hgb followed by NLR and SII providing the most improvement in AUROC. For 90-day mortality alone, NLR provided the most improvement in discrimination, followed by ANC, SII, and Hgb. For 90-day all-cause and causes specific rehospitalizations, Hgb consistently improved discrimination as compared to all other CBCD parameters. To further evaluate the relationship of Hgb with the primary outcome, sub-group analysis was performed excluding patients with cancer, baseline anemia, or liver disease. Exclusion of these patients had minimal effect on the added value of Hgb in predicting the primary outcome (**Supplemental Table 11**).

**Table 3:** Discrimination using area under the receiver operator characteristics (AUROC) curves to determine the added value of CBCD parameters to clinical charactersitics in predicting 90-day outcome.

Model	90-day mortality or rehospitalization	90-day mortality	90-day rehospitalization (all-cause)	90-day rehospitalization (potential infection)	90-day rehospitalization (sepsis)
**Base, AUROC**	0.6756	0.7590	0.6318	0.6289	0.6381
**Full Model, AUROC (ΔAUC)**	0.6929 **(+0.0173)**	0.7936 **(+0.0346)**	0.6406 **(+0.0088)**	0.6372 **(+0.0083)**	0.6503 **(+0.0122)**
Base + individual CBCD, AUROC (ΔAUC)					
** + Hgb**	0.6840 **(+0.0084)**	0.7673 **(+0.0083)**	0.6386 **(+0.0068)**	0.6322 **(+0.0033)**	0.6445 **(+0.0064)**
** + NLR**	0.6823 **(+0.0067)**	0.7772 **(+0.0182)**	0.6323 **(+0.0005)**	0.6296 **(+0.0007)**	0.6388 **(+0.0007)**
** + SII**	0.6798 **(+0.0042)**	0.7678 **(+0.0088)**	0.6323 **(+0.0005)**	0.6299 **(+0.0010)**	0.6392 **(+0.0011)**
** + ANC**	0.6793 **(+0.0037)**	0.7698 **(+0.0108)**	0.6322 **(+0.0004)**	0.6307 **(+0.0018)**	0.6403 **(+0.0022)**
** + ALC**	0.6792 **(+0.0036)**	0.7666 **(+0.0076)**	0.6324 **(+0.0006)**	0.6300 **(+0.0011)**	0.6386 **(+0.0005)**
** + WBC**	0.6781 **(+0.0025)**	0.7658 **(+0.0068)**	0.6323 **(+0.0005)**	0.6313 **(+0.0024)**	0.6405 **(+0.0024)**
** + Plt**	0.6779 **(+0.0023)**	0.7656 **(+0.0066)**	0.6326 **(+0.0008)**	0.6306 **(+0.0017)**	0.6414 **(+0.0033)**
** + PLR**	0.6774 **(+0.0018)**	0.7621 **(+0.0031)**	0.6324 **(+0.0006)**	0.6298 **(+0.0009)**	0.6388 **(+0.0007)**

Clinical (Base) model: logistic regression predicting 90-day outcome based on clinical characteristics including age, sex, individual comorbid conditions, acute organ dysfunction count, ICU use, and length of hospitalization.

Full Model: Base model + all 8 CBCD parameters.

All models compared to base model using the Likelihood-ratio test: -2ln(likelihood of base model/likelihood base + CBCD model), p-value < 0.02 for all tests.

ΔAUC, delta area under the curve = AUROC_Base+CBCD_ – AUROC_Base_. WBC, total white blood cell count; Hgb, hemoglobin; Plt, platelet count; ANC, absolute neutrophil count; ALC, absolute lymphocyte count; NLR, neutrophil-to-lymphocyte ratio (NLR = ANC/ALC); PLR, platelet-to-lymphocyte ratio (PLR = Plt/ALC); SII, systemic immune-inflammation index (SII = ANC x PLR/ALC).

To determine if CBCD parameters significantly improved model performance, we utilized the likelihood ratio test (LRT) comparing all models with CBCD parameters against the base model (**Supplemental Table 12**). Model fit was significantly improved among models including CBCD parameters for all outcomes (all LRT p<0.02). We then compared likelihood ratio χ^2^ values to determine the added value of each parameter to the base model. As expected, the full model had the highest χ^2^ values for all outcomes. Among individual CBCD parameters, Hgb and NLR had the highest χ^2^ values for the primary outcome. For 90-day mortality alone, NLR and ANC had the highest χ^2^ values followed again by SII and Hgb. For 90-day rehospitalizations, Hgb had the highest χ^2^ values for both all-cause and cause-specific outcome.

## Discussion

In this nationwide cohort of more than 150,000 sepsis hospitalizations at 138 hospitals, derangements in CBCD parameters at sepsis discharge were common and associated with increased risk for 90-day mortality or rehospitalization. The most common CBCD abnormalities were anemia, lymphopenia, thrombocytopenia, and leukocytosis/neutrophilia. In univariable survival analysis, hemoglobin had the greatest prognostic separation for the primary outcome and all secondary outcomes. In a subset of more than 80,000 hospitalizations, multivariable models including clinical characteristics with or without CBCD parameters were compared. The addition of all eight CBCD parameters to clinical characteristics resulted in significant improvement in discrimination for predicting 90-day outcome. The addition of CBCD parameters reduced model mean calibration error by 50%, contributing to appropriate reclassification of predicted risk for every 1 in 100 hospitalizations. Individual parameters were evaluated and marginally improved discrimination. Hgb and NLR provided the most added information in predicting the primary outcome, whereas NLR and ANC provided the most added information in predicting 90-day mortality alone. As compared to other CBCD parameters, Hgb provided the most added value in predicting all-cause and cause-specific rehospitalization for new infection/sepsis.

Prior to this work, there was little data broadly characterizing CBCD parameters measured at discharge and their relationship with longer-term outcomes among sepsis survivors. This is also the first report of the relative value of leukocyte and non-leukocyte parameters measured at hospital discharge in prediction of long-term outcomes in sepsis survivors. Predicting risk for long-term complications, particularly rehospitalization, is a complex task. The addition of CBCD parameters to clinical parameters improved risk prediction, though full model performance was overall fair to good (AUROC, 0.693). This is similar to a previously reported prediction model for 1-year rehospitalization and death in sepsis survivors (AUROC, 0.675) (8). This prior model also included measures of socioeconomic status and preceding healthcare utilization which were not included in our base model. Though some of these more complex clinical variables may be useful for prediction, they can be difficult to obtain or easily implement. Therefore, discharge CBCD parameters may have additional value for prediction in their simplicity and ready availability. Among CBCD parameters, we found that Hgb was more useful than leukocyte parameters in predicting certain long-term outcomes in sepsis survivors. This is a novel observation that has implications for the value of incorporating this biological variable in future risk prediction models.

Several retrospective studies have identified Hgb as an important prognostic biomarker in sepsis survivors when measured on hospital admission or discharge (8, 37, 40–44). Among survivors of sepsis and critical illness, lower discharge Hgb is an independent risk factor for 30-day rehospitalization and posthospitalization mortality (37, 40, 41, 44, 45). Among patients discharged with anemia after critical illness, impaired recovery of Hgb is also associated with higher rates of 1-year rehospitalization and death (43). Our study further identifies that discharge Hgb, as compared to leukocyte parameters, adds more predictive value for 90-day all-cause and infection-related rehospitalization in sepsis survivors. The intent of this study was to evaluate the additive value of several CBCD parameters in risk prediction, as such we did not specifically account for potential confounding causes of anemia (e.g., transfusions received, blood draws) in our clinical model. However, common clinical causes of anemia (e.g., renal disease, blood loss anemia, and deficiency anemia) were accounted for in the base model within the co-morbid conditions. Inflammatory anemia is also a common cause of anemia in hospitalized individuals and is more prevalent among those with elevated circulating inflammatory markers, such as C-reactive protein (CRP) (46, 47). Persistent elevation of CRP is observed in some sepsis survivors and is associated with increased risk for rehospitalization and/or death (12). Taken together, a link between discharge anemia, inflammation, and host response to infection is suggested. Examining the role of inflammatory anemia, iron metabolism, and the host response to secondary infections after sepsis could be considered in future translational work.

Lymphopenia is a hallmark of acute sepsis, and severe lymphopenia is associated with increased risk for in-hospital mortality and secondary nosocomial infections (19, 24, 33, 48–50). The most common reason for rehospitalization in sepsis survivors is new infection or sepsis (2, 51). Though lymphopenia occurred in nearly one-third of sepsis survivors, it added the least additional value to clinical characteristics in predicting 90-day rehospitalizations due to new sepsis. The strongest candidate leukocyte parameter was NLR in predicting the primary outcome and 90-day mortality alone. However, similar to ALC, NLR added little value in predicting risk for rehospitalization. NLR did have improved predictive ability across multiple outcomes as compared to ANC and ALC alone, suggesting that combination parameters provide more prognostic information than individual parameters alone.

This study provides new data on the prevalence of CBCD derangements and their impact on predicting long-term outcomes. As candidate biomarkers, CBCD parameters are inexpensive, readily available, and can be easily incorporated into prognostic scoring systems. Multivariable models utilizing discharge CBCD parameters may be most useful to improve model discrimination and calibration when complex clinical variables are not easily obtained. Additional work will be required to validate the utility of these parameters in this capacity. This study also adds to several large studies (44, 45) showing that Hgb is an important predictor of long-term outcomes among survivors of sepsis and/or critical illness. However, as our study does not evaluate causation, additional work is needed to determine if anemia at hospital discharge contributes directly to long-term outcomes.

There are several limitations of this study. First, this cohort was predominantly male and White, so generalizability to other populations is unclear. Future studies with greater sex and racial representation are needed to evaluate these relationships further. Second, as our primary objective was to explore the relationship of multiple parameters with multiple outcomes, we did not perform external validation for the variables or internal validation with dataset splitting which might limit the generalizability of our predictive model. While we carefully selected variables that had been validated in previously published models in sepsis survivors in other healthcare settings, our findings should be interpreted as the incremental impact of CBCD parameters under optimistic conditions. Third, we intentionally trimmed the top and bottom 1% of values from each CBCD parameter to exclude individuals with bone marrow failure or malignancy and this may have weakened the strength of associations measured. Finally, outcomes were extracted from the electronic health record. While mortality was fully captured, we were not able to capture rehospitalization outside the VA.

## Conclusion

In this national cohort of sepsis survivors, derangements in CBCD parameters at discharge were common and associated with increased risk for 90-day mortality and rehospitalization. Hgb most consistently added value to clinical characteristics in predicting 90-day outcomes, including rehospitalizations related to infection. NLR provided the most added value in predicting 90-day mortality. The incorporation of discharge CBCD parameters into prognostic scoring systems could improve prediction of 90-day outcomes in sepsis survivors.

## Data availability statement

The original contributions presented in the study are included in the article/**Supplementary Materials**, further inquiries can be directed to the corresponding author/s.

## Ethics statement

The studies involving human participants were reviewed and approved by Ann Arbor VA Institutional Review Board. Written informed consent for participation was not required for this study in accordance with the national legislation and the institutional requirements.

## Author contributions

SD and HP designed the study. SD, JC, XW, SS, and HP collected the data. SD, JC, JD, SS, and HP analyzed and interpreted the data. SD and HP drafted the manuscript. All author provided critical revision and approval of the final version of the manuscript.
