# Supplementary material for: Blood count derangements after sepsis and association with post-hospital outcomes
Source: Front Immunol. 2023 Feb 28;14:1133351. doi: 10.3389/fimmu.2023.1133351 (PMC10018394; doi:10.3389/fimmu.2023.1133351)
Supplement: Supplementary file 1 [file DataSheet_1.docx]

**ONLINE DATA SUPPLEMENT**

**Title:** Blood count derangements after sepsis and association with post-hospital outcomes **Authors:** Scott J. Denstaedt, Jennifer Cano, Xiao Qing Wang, John P. Donnelly, Sarah Seelye, Hallie C. Prescott

| **Table of Contents** | | |
| --- | --- | --- |
| **Supplemental Methods** | R packages and versions | Page 3 |
| **Supplemental Table 1** | Elixhauser comorbidities | Page 3 |
| **Supplemental Table 2** | Discharge CBCD lab data after exclusion of non-physiologic results | Page 4 |
| **Supplemental Table 3** | Discharge CBCD lab data after exclusion of top and bottom 1% following exclusion of non-physiologic results | Page 5 |
| **Supplemental Table 4** | Characteristics of sepsis cohort, extended | Page 6 |
| **Supplemental Table 5** | Abnormal CBCD parameters among patients with hospitalization for sepsis and live discharge | Page 8 |
| **Supplemental Table 6** | Characteristics of sepsis cohort – all-cause 90-day mortality, 90-day rehospitalization | Page 9 |
| **Supplemental Table 7** | Characteristics of patients readmitted within 90-days by cause of rehospitalization | Page 12 |
| **Supplemental Figure 1** | Association of combination CBCD parameters and cumulative incidence of 90-day mortality or rehospitalization | Page 14 |
| **Supplemental Table 8** | Characteristics of patients included/excluded for the base model | Page 15 |
| **Supplemental Figure 2** | Calibration plots for Base and Full model by 90-day outcome | Page 17 |
| **Supplemental Table 9** | Observed vs predicted mortality using 10 equally-size bins defined by decile of predicted risk using the Base model | Page 19 |
| **Supplemental Table 10** | Observed vs predicted mortality using 10 equally-size bins defined by decile of predicted risk using the Full model | Page 20 |
| **Supplemental Table 11** | Sub-group analysis based on baseline co-morbid disease | Page 21 |
| **Supplemental Table 12** | Likelihood ratio chi-squares for models including CBCD parameters as compared to the Base model for predicting 90-day outcome | Page 22 |

**R packages:** *tidyverse*, version 1.3.1; *survival*, version 3.3-1; *rms*, version 6.2-0; *pROC*, version 1.18.0, *lmtest*; version 0.9-40, *eulerr*; version 6.1.1; *ggplot2*, version 3.3.5.

| Supplemental Table 1. Elixhauser comorbidities based on International Classification of Disease (ICD) codes extracted from the medical record | |
| --- | --- |
| Organ system | **Elixhauser comorbidity** |
| Cardiac | Congestive Heart Failure |
|  | Cardiac Arrhythmia |
|  | Valvular Disease |
|  | Pulmonary Circulation Disorders |
|  | Peripheral Vascular Disorders |
|  | Hypertension Complicated and Uncomplicated |
| Neuropsychiatric | Paralysis |
|  | Psychosis |
|  | Depression |
|  | Other Neurological Disorders |
| Pulmonary | Chronic Pulmonary Disease |
| Endocrine | Diabetes Uncomplicated |
|  | Diabetes Complicated |
|  | Hypothyroidism |
| Renal | Renal Failure |
| GI/Liver | Liver Disease |
|  | Peptic Ulcer Disease excluding bleeding |
| Immunodeficiency | AIDS/HIV |
| Malignancy | Lymphoma |
|  | Metastatic Cancer |
|  | Solid Tumor without Metastasis |
| Rheumatic | Rheumatoid Arthritis/collagen |
| Hematologic | Coagulopathy |
|  | Blood Loss Anemia |
|  | Deficiency Anemia |
| Alcohol/Drug use | Alcohol Abuse |
|  | Drug Abuse |
| Other | Obesity |
|  | Weight Loss |
|  | Fluid and Electrolyte Disorders |

| **Supplemental Table 2: Discharge CBCD lab data after exclusion of non-physiologic* results** | | | | | | | | | | |
| --- | --- | --- | --- | --- | --- | --- | --- | --- | --- | --- |
| **CBC Parameter** | **Total**  **measurements** | **% of live discharge** | **Mean** | **Min** | **1^st^**  **%ile** | **25^th^**  **%ile** | **Median** | **75^th^**  **%ile** | **99^th^**  **%ile** | **Max** |
| WBC (10^3^/µL) | 155,374 | 84.7% | 9.21 | 0.0001 | 1.50 | 6.11 | 8.40 | 11.0 | 26.10 | 293.1 |
| Hemoglobin (g/dL) | 155,960 | 85.1% | 10.83 | 4.00 | 7.20 | 9.20 | 10.70 | 12.30 | 15.80 | 21.80 |
| Platelet count (10^3^/µL) | 155,031 | 84.5% | 232.8 | 10.00 | 26.00 | 150.0 | 215.0 | 294.0 | 629.0 | 1483.0 |
| ANC (10^3^/µL) | 102,890 | 56.1% | 6.28 | 0.01 | 0.13 | 3.80 | 5.65 | 7.97 | 18.55 | 293.0 |
| ALC (10^3^/µL) | 106,491 | 58.1% | 1.63 | 0.01 | 0.21 | 0.90 | 1.30 | 1.88 | 4.83 | 273.1 |
| Distribution of discharge CBCD parameters after excluding non-physiologic values.  *We considered the following thresholds to define non-physiologic values: WBC 0 or >300, hemoglobin <1 or >24, platelet <10 or >1500, ANC 0 or >300, ALC 0 or >300.  WBC, total white blood cell count. ANC, absolute neutrophil count. ALC, absolute lymphocyte count. | | | | | | | | | | |

| **Supplemental Table 3: Discharge CBCD lab data after exclusion of top and bottom 1% following exclusion of non-physiologic results** | | | | | | | | | | |
| --- | --- | --- | --- | --- | --- | --- | --- | --- | --- | --- |
| **CBC Parameter** | **Total**  **measurements** | **% of live discharge** | **Mean** | **Min** | **1^st^**  **%ile** | **25^th^**  **%ile** | **Median** | **75^th^**  **%ile** | **99^th^**  **%ile** | **Max** |
| WBC (10^3^/µL) | 152,415 | 83.1% | 8.85 | 1.50 | 2.10 | 6.20 | 8.39 | 10.90 | 21.20 | 26.10 |
| Hemoglobin (g/dL) | 153,022 | 83.4% | 10.82 | 7.20 | 7.40 | 9.20 | 10.70 | 12.2 | 15.2 | 15.80 |
| Platelet count (10^3^/µL) | 152,030 | 82.9% | 229.7 | 26.00 | 37.00 | 152.0 | 214.0 | 292.0 | 558.0 | 629.0 |
| ANC (10^3^/µL) | 100,840 | 55.0% | 6.14 | 0.13 | 0.70 | 3.80 | 5.65 | 7.90 | 15.99 | 18.55 |
| ALC (10^3^/µL) | 104,385 | 56.9% | 1.45 | 0.21 | 0.30 | 0.90 | 1.30 | 1.85 | 3.80 | 4.83 |
| NLR | 92,305 | 50.3% | 5.48 | 0.04 | 0.63 | 2.59 | 4.11 | 6.64 | 24.91 | 85.05 |
| PLR | 95,027 | 51.8% | 192.8 | 6.09 | 35.27 | 109.3 | 160.8 | 237.8 | 684.3 | 2540.9 |
| SII | 84,198 | 45.9% | 1253.6 | 1.22 | 61.22 | 490.0 | 888.3 | 1560.0 | 6355.0 | 30624.3 |
| Distribution of discharge CBCD parameters after excluding the top and bottom 1% of values for WBC, Hemoglobin, Platelet count, ANC, and ALC.  WBC, total white blood cell count. ANC, absolute neutrophil count. ALC, absolute lymphocyte count. | | | | | | | | | | |

| **Supplemental Table 4. Characteristics of Sepsis Cohort, stratified by composite outcome** | | | | |
| --- | --- | --- | --- | --- |
| **Patient Characteristics** | **Total cohort**  **(n = 155,988)** | **No mortality or rehospitalization**  **(n = 86926)** | **90-day all-cause mortality or rehospitalization**  **(n = 69062)** | **P value** |
| **Age, median (IQR)** | 68 (62-76) | 68 (61-75) | 69 (63-77) | <0.001 |
| **Sex, M, %** | 96.3 | 96.0 | 96.8 | <0.001 |
| **Race, %** |  |  |  |  |
| Black | 20.9 | 20.6 | 21.2 | <0.001 |
| White | 71.4 | 71.4 | 71.3 |  |
| Other | 2.2 | 2.3 | 2.0 |  |
| Unknown | 5.4 | 5.5 | 5.3 |  |
| **Comorbidity count, median (IQR)** | 6 (4-8) | 5 (3-7) | 7 (5-9) | <0.001 |
| **Select comorbid conditions, n (%)** |  |  |  |  |
| Any Diabetes | 79024 (50.7) | 42884 (49.3) | 36140 (52.3) | <0.001 |
| Chronic pulmonary disease | 72977 (46.8) | 38227 (44.0) | 34750 (50.3) | <0.001 |
| CKD | 55482 (35.6) | 26717 (30.7) | 28765 (41.7) | <0.001 |
| Diabetes with complication | 52399 (33.6) | 27538 (31.7) | 24861 (36.0) | <0.001 |
| Congestive heart failure | 51209 (32.8) | 23787 (27.4) | 27422 (39.7) | <0.001 |
| Any Cancer | 42048 (27.0) | 18959 (21.8) | 23089 (33.4) | <0.001 |
| Liver disease | 29595 (19.0) | 13947 (16.0) | 15648 (22.7) | <0.001 |
| Neurologic disease | 27558 (17.7) | 13046 (15.0) | 14512 (21.0) | <0.001 |
| Metastatic cancer | 12107 (7.8) | 4088 (4.7) | 8019 (11.6) | <0.001 |
| Rheumatoid Arthritis/Collagen Vascular Diseases | 6872 (4.4) | 3516 (4.0) | 3356 (4.9) | <0.001 |
| **Acute organ dysfunction count, median (IQR)** | 1 (1-2) | 1 (1-2) | 1 (1-2) | <0.001 |
| **Acute organ dysfunctions, n (%)** |  |  |  |  |
| Renal | 95367 (61.1) | 51441 (59.2) | 43926 (63.6) | <0.001 |
| Elevated lactate | 68505 (43.9) | 39234 (45.1) | 29271 (42.4) | <0.001 |
| Hematologic | 20501 (13.1) | 8942 (10.3) | 11559 (16.7) | <0.001 |
| Hepatic | 20192 (12.9) | 10718 (12.3) | 9474 (13.7) | <0.001 |
| Circulatory (Shock) | 11827 (7.6) | 6180 (7.1) | 5647 (8.2) | <0.001 |
| Mechanical ventilation | 7080 (4.5) | 3555 (4.1) | 3525 (5.1) | <0.001 |
| **ICU admission, n (%)** | 46061 (29.5) | 23274 (26.8) | 22787 (33.0) | <0.001 |
| **Any surgery during hospitalization** | 20,996 (13.5) | 12,112 (13.9) | 8884 (12.9) | <0.001 |
| **Hospital LOS, median (IQR)** | 6 (4-10) | 6 (4-9) | 7 (5-11) | <0.001 |
| **Discharge blood parameter, median (IQR)** |  |  |  |  |
| WBC (10^3^/µL) | 8.39 (6.20-10.90) | 8.31 (6.30-10.80) | 8.40 (6.09-11.10) | 0.24 |
| Hemoglobin (g/dL) | 10.7 (9.2-12.20) | 11.2 (9.70-12.6) | 10.1 (8.80-11.7) | <0.001 |
| Platelet count (10^3^/µL) | 214 (152-292) | 219 (160-294) | 208 (139-289) | <0.001 |
| ANC (10^3^/µL) | 5.65 (3.80-7.90) | 5.6 (3.83-7.71) | 5.72 (3.80-8.10) | <0.001 |
| ALC (10^3^/µL) | 1.3 (0.90-1.85) | 1.4 (1.00-1.90) | 1.2 (0.80-1.73) | <0.001 |
| NLR | 4.11 (2.59-6.64) | 3.86 (2.48-6.12) | 4.48 (2.77-7.40) | <0.001 |
| PLR | 161 (109-238) | 156 (108-227) | 167.59 (111-252) | <0.001 |
| SII | 888 (490-1560) | 851(484-1462) | 948.23 (501-1701) | <0.001 |
| Comorbidity count determined using 30 Elixhauser comorbidities.  P-value calculated comparing those with and without the primary composite outcome.  WBC, total white blood cell count. ANC, absolute neutrophil count. ALC, absolute lymphocyte count. NLR, neutrophil-to-lymphocyte ratio (NLR = ANC / ALC). PLR, platelet-to-lymphocyte ratio (PLR = Plt / ALC). SII, systemic immune-inflammation index (SII = ANC x PLR / ALC). | | | | |

| **Supplemental Table 5. Abnormal CBCD parameters among patients with hospitalization for sepsis and live discharge** | | | | |
| --- | --- | --- | --- | --- |
| **CBC Parameter** | **N** | **Normal range** | **Below normal range**  **N (%)** | **Above normal range**  **N (%)** |
| WBC (10^3^/µL) | 152,415 | 4.0 – 11.0 | 11,566 (7.6) | 36,534 (24.0) |
| Hemoglobin (g/dL) | 153,022 | 14.0 – 18.0 | 143,162 (93.6) | 0 (0.0) |
| Platelet count (10^3^/µL) | 152,030 | 150.0 – 400.0 | 36,817 (24.2) | 12,175 (8.0) |
| ANC (10^3^/µL) | 100,819 | 1.50 – 8.00 | 3,586 (3.6) | 24,072 (23.9) |
| ALC (10^3^/µL) | 104,352 | 1.00 – 4.00 | 29,365 (28.1) | 685 (0.7) |
| Reported normal ranges varied slightly across individual VA hospitals. Here we defined the normal range cut-offs specific to each parameter using the mode of reference values across all VA hospitals.  WBC, total white blood cell count. Hgb, hemoglobin. ANC, absolute neutrophil count. ALC, absolute lymphocyte count. | | | | |

| **Supplemental Table 6.** **Characteristics of sepsis cohort – all-cause 90-day mortality, 90-day rehospitalization** | | | | | | |
| --- | --- | --- | --- | --- | --- | --- |
|  | **Survived**  **(n = 132,920)** | **Died**  **(n = 23,068)** | **P value** | **Not rehospitalized**  **(n = 100,928)** | **Rehospitalized**  **(n = 55,060)** | **P value** |
| **Age, median (IQR)** | 68 (61-75) | 72 (65-83) | <0.001 | 68 (62-76) | 68 (62-75) | <0.001 |
| **Sex, M, %** | 127765 (96.1) | 22520 (97.6) | <0.001 | 97107 (96.2) | 53178 (96.6) | 0.002 |
| **Race, %** |  |  |  |  |  |  |
| Black | 27912 (21.0) | 4677 (20.3) | <0.001 | 20704 (20.5) | 11885 (21.6) | <0.001 |
| White | 94852 (71.4) | 16487 (71.5) |  | 72141 (71.5) | 39198 (71.2) |  |
| Other | 2988 (2.2) | 409 (1.8) |  | 2250 (2.2) | 1147 (2.1) |  |
| Unknown | 7023 (5.3) | 1436 (6.2) |  | 5682 (5.6) | 2777 (5.0) |  |
| **Comorbidity count, median (IQR)** | 6 (4-9) | 8 (6-10) | <0.001 | 6 (4-8) | 8 (5-10) | <0.001 |
| **Select comorbid conditions, n (%)** |  |  |  |  |  |  |
| Any Diabetes | 68076 (51.2) | 10948 (47.5) | <0.001 | 49202 (48.7) | 29822 (54.2) | <0.001 |
| Chronic pulmonary disease | 61138 (46.0) | 11839 (51.3) | <0.001 | 45270 (44.9) | 27707 (50.3) | <0.001 |
| CKD | 45692 (34.4) | 9790 (42.4) | <0.001 | 32464 (32.2) | 23018 (41.8) | <0.001 |
| Diabetes with complication | 45206 (34.0) | 7193 (31.2) | <0.001 | 31633 (31.3) | 20766 (37.7) | <0.001 |
| Congestive heart failure | 41682 (31.4) | 9527 (41.3) | <0.001 | 29428 (29.2) | 21781 (39.6) | <0.001 |
| Any Cancer | 31715 (23.9) | 10333 (44.8) | <0.001 | 25241 (25.0) | 16807 (30.5) | <0.001 |
| Liver disease | 24136 (18.2) | 5459 (23.7) | <0.001 | 16980 (16.8) | 12615 (22.9) | <0.001 |
| Neurologic disease | 22123 (16.6) | 5435 (23.6) | <0.001 | 16486 (16.3) | 11072 (20.1) | <0.001 |
| Metastatic cancer | 7362 (5.5) | 4745 (20.6) | <0.001 | 7118 (7.1) | 4989 (9.1) | <0.001 |
| Rheumatoid Arthritis/Collagen Vascular Diseases | 5850 (4.4) | 1022 (4.4) | 0.842 | 4118 (4.1) | 2754 (5.0) | <0.001 |
| **Acute organ dysfunction count, median (IQR)** | 1 (1-2) | 1 (1-2) | <0.001 | 1 (1-2) | 1 (1-2) | <0.001 |
| **Acute organ dysfunctions, n (%)** |  |  |  |  |  |  |
| Renal | 80647 (60.7) | 14720 (63.8) | <0.001 | 60496 (59.9) | 34871 (63.3) | <0.001 |
| Elevated lactate | 58035 (43.7) | 10470 (45.4) | <0.001 | 45900 (45.5) | 22605 (41.1) | <0.001 |
| Hematologic | 15832 (11.9) | 4669 (20.2) | <0.001 | 11545 (11.4) | 8956 (16.3) | <0.001 |
| Hepatic | 16596 (12.5) | 3596 (15.6) | <0.001 | 12860 (12.7) | 7332 (13.3) | 0.001 |
| Circulatory (Shock) | 9582 (7.2) | 2245 (9.7) | <0.001 | 7651 (7.6) | 4176 (7.6) | 0.978 |
| Respiratory | 5709 (4.3) | 1371 (5.9) | <0.001 | 4464 (4.4) | 2616 (4.8) | 0.003 |
| **ICU admission, n (%)** | 37254 (28.0) | 8807 (38.2) | <0.001 | 29010 (28.7) | 17051 (31.0) | <0.001 |
| **Hospital LOS, median (IQR)** | 6 (4-9) | 8 (5-12) | <0.001 | 6 (4-9) | 7 (5-11) | <0.001 |
| **Discharge blood parameter, median (IQR)** |  |  |  |  |  |  |
| WBC (10^3^/µL) | 8.30 (6.18-10.74) | 8.90 (6.37-12.10) | <0.001 | 8.45 (6.30-11.00) | 8.2 (5.90-10.80) | <0.001 |
| Hemoglobin (g/dL) | 10.90 (9.40-12.40) | 9.60 (8.60-11.10) | <0.001 | 11 (9.50-12.50) | 10.20 (8.90-11.80) | <0.001 |
| Platelet count (10^3^/µL) | 218 (156-294) | 193 (120-279) | <0.001 | 216 (156-292) | 211 (143-291) | <0.001 |
| ANC (10^3^/µL) | 5.56 (3.80-7.70) | 6.38 (4.20-9.10) | <0.001 | 5.70 (3.90-7.99) | 5.50 (3.70-7.76) | <0.001 |
| ALC (10^3^/µL) | 1.39 (0.97-1.90) | 1.10 (0.70-1.53) | <0.001 | 1.36 (0.94-1.90) | 1.30 (0.88-1.80) | <0.001 |
| NLR | 3.93 (2.50-6.24) | 5.74 (3.46-9.50) | <0.001 | 4.06 (2.57-6.59) | 4.20 (2.63-6.73) | <0.001 |
| PLR | 158 (109-232) | 180 (115-278) | <0.001 | 159 (109-234) | 164 (110-245) | <0.001 |
| SII | 860 (481-1486) | 1132 (579-2100) | <0.001 | 885 (496-1547) | 896 (477-1585) | 0.633 |
| Comorbidity count determined using 30 Elixhauser comorbidities.  P-value calculated comparing those with and without either mortality or rehospitalization.  WBC, total white blood cell count. ANC, absolute neutrophil count. ALC, absolute lymphocyte count. NLR, neutrophil-to-lymphocyte ratio (NLR = ANC / ALC). PLR, platelet-to-lymphocyte ratio (PLR = Plt / ALC). SII, systemic immune-inflammation index (SII = ANC x PLR / ALC). | | | | | | |

| **Supplemental Table 7. Characteristics of patients readmitted within 90-days by cause of rehospitalization** | | | |
| --- | --- | --- | --- |
|  | **90-day all cause rehospitalization**  **(n = 55,060)** | **90-day rehospitalization**  **potential infection**  **(n = 32,665)** | **90-day rehospitalization sepsis**  **(n = 13,802)** |
| **Age, median (IQR)** | 68 (62-75) | 68 (62-76) | 68 (62-76) |
| **Sex, M, %** | 53178 (96.6) | 31521 (96.5) | 13348 (96.7) |
| **Race, %** |  |  |  |
| Black | 11885 (21.6) | 7130 (21.8) | 3027 (21.9) |
| White | 39198 (71.2) | 23237 (71.1) | 9776 (70.8) |
| Other | 1147 (2.1) | 664 (2.0) | 315 (2.3) |
| Unknown | 2777 (5.0) | 1604 (4.9) | 675 (4.9) |
| **Comorbidity count, median (IQR)** | 8 (5-10) | 8 (5-10) | 8 (6-10) |
| **Select comorbid conditions, n (%)** |  |  |  |
| Any Diabetes | 29822 (54.2) | 17393 (53.2) | 7549 (54.7) |
| Chronic pulmonary disease | 27707 (50.3) | 17908 (54.8) | 7193 (52.1) |
| CKD | 23018 (41.8) | 13650 (41.8) | 6204 (45.0) |
| Diabetes with complication | 20766 (37.7) | 11896 (36.4) | 5213 (37.8) |
| Congestive heart failure | 21781 (39.6) | 13333 (40.8) | 5437 (39.4) |
| Any Cancer | 16807 (30.5) | 10325 (31.6) | 4691 (34.0) |
| Liver disease | 12615 (22.9) | 7575 (23.2) | 3371 (24.4) |
| Neurologic disease | 11072 (20.1) | 6772 (20.7) | 3035 (22.0) |
| Metastatic cancer | 4989 (9.1) | 3222 (9.9) | 1452 (10.5) |
| Rheumatoid Arthritis/Collagen Vascular Diseases | 2754 (5.0) | 1707 (5.2) | 784 (5.7) |
| **Acute organ dysfunction count, median (IQR)** | 1 (1-2) | 1 (1-2) | 1 (1-2) |
| **Acute organ dysfunctions, n (%)** |  |  |  |
| Renal | 34871 (63.3) | 20209 (61.9) | 8932 (64.7) |
| Elevated lactate | 22605 (41.1) | 13670 (41.8) | 5622 (40.7) |
| Hematologic | 8956 (16.3) | 5551 (17.0) | 2788 (20.2) |
| Hepatic | 7332 (13.3) | 4043 (12.4) | 1803 (13.1) |
| Circulatory (Shock) | 4176 (7.6) | 2363 (7.2) | 974 (7.1) |
| Respiratory | 2616 (4.8) | 1588 (4.9) | 616 (4.5) |
| **ICU admission, n (%)** | 17051 (31.0) | 10034 (30.7) | 4173 (30.2) |
| **Hospital LOS, median (IQR)** | 7 (5-11) | 7 (5-10) | 7 (5-11) |
| **Discharge blood parameter, median (IQR)** |  |  |  |
| WBC (10^3^/µL) | 8.2 (5.90-10.80) | 8.3 (5.90-11.00) | 8.26 (5.70-10.97) |
| Hemoglobin (g/dL) | 10.20 (8.90-11.80) | 10.2 (8.90-11.80) | 9.90 (8.70-11.40) |
| Platelet count (10^3^/µL) | 211 (143-291) | 209 (140-288) | 204 (130-286) |
| ANC (10^3^/µL) | 5.50 (3.70-7.76) | 5.6 (3.66-7.93) | 5.50 (3.50-7.90) |
| ALC (10^3^/µL) | 1.30 (0.88-1.80) | 1.28 (0.83-1.80) | 1.24 (0.81-1.80) |
| NLR | 4.20 (2.63-6.73) | 4.28 (2.66-6.88) | 4.25 (2.62-6.85) |
| PLR | 164 (110-245) | 163 (109-245) | 163 (108-245) |
| SII | 896 (477-1585) | 907.38 (477-1626) | 904 (459-1612) |
| Comorbidity count determined using 30 Elixhauser comorbidities.  WBC, total white blood cell count. ANC, absolute neutrophil count. ALC, absolute lymphocyte count. NLR, neutrophil-to-lymphocyte ratio (NLR = ANC / ALC). PLR, platelet-to-lymphocyte ratio (PLR = Plt / ALC). SII, systemic immune-inflammation index (SII = ANC x PLR / ALC). | | | |

**Supplemental Figure 1. Association of combination CBCD parameters and cumulative incidence of 90-day mortality or rehospitalization.** Univariate Kaplan-Meier failure plots showing cumulative incidence of the primary outcome by quintile. Neutrophil Lymphocyte Ratio = absolute neutrophil count / absolute lymphocyte count. Platelet Lymphocyte Ratio = platelet count / absolute lymphocyte count. Systemic Immune-Inflammation index = (absolute neutrophil count x platelet count) / absolute lymphocyte count.


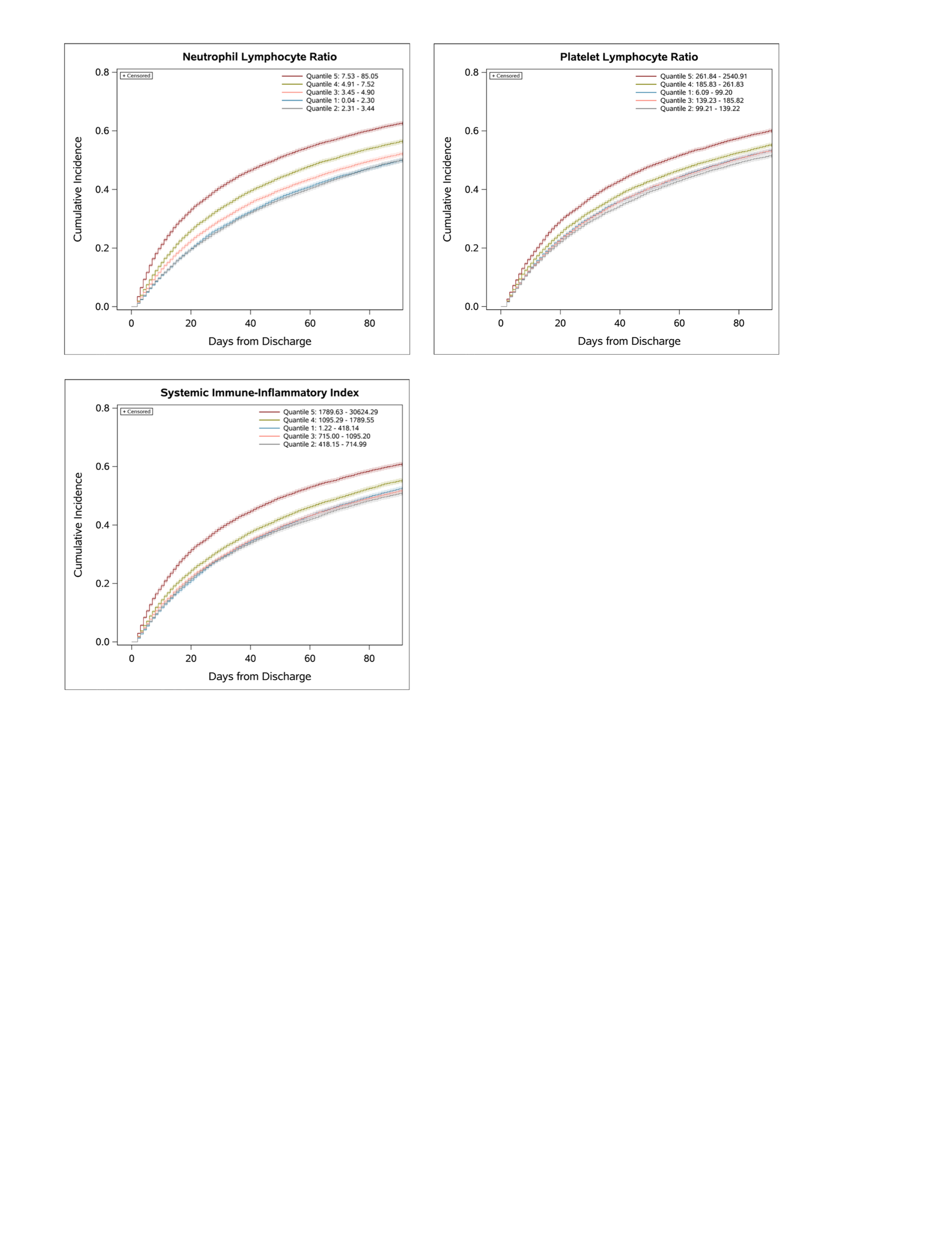


| **Supplemental Table 8.** **Characteristics of patients included/excluded for base model** | | | |
| --- | --- | --- | --- |
|  | **Included in Base model (N = 82,510)** | **Excluded from Base model (N = 73,478)** | **P value** |
| **Age, median (IQR)** | 68 (62-77) | 68 (62-75) | <0.001 |
| **Sex, M, %** | 79538 (96.4) | 70747 (96.3) | 0.228 |
| **Race, %** |  |  |  |
| Black | 16824 (20.4) | 15765 (21.5) | <0.001 |
| White | 59191 (71.7) | 52148 (71.0) |  |
| Other | 1733 (2.1) | 1664 (2.3) |  |
| Unknown | 4638 (5.6) | 3821 (5.2) |  |
| **Comorbidity count, median (IQR)** | 7 (4-9) | 7 (4-9) | 0.422 |
| **Select comorbid conditions, n (%)** |  |  |  |
| Any Diabetes | 42577 (51.6) | 36447 (49.6) | <0.001 |
| Chronic pulmonary disease | 39298 (47.6) | 33679 (45.8) | <0.001 |
| CKD | 29577 (35.8) | 25905 (35.3) | 0.015 |
| Diabetes with complication | 28589 (34.6) | 23810 (32.4) | <0.001 |
| Congestive heart failure | 27044 (32.8) | 24165 (32.9) | 0.642 |
| Any Cancer | 21137 (25.6) | 20911 (28.5) | <0.001 |
| Liver disease | 15212 (18.4) | 14383 (19.6) | <0.001 |
| Neurologic disease | 14993 (18.2) | 12565 (17.1) | <0.001 |
| Metastatic cancer | 5813 (7.0) | 6294 (8.6) | <0.001 |
| Rheumatoid Arthritis/Collagen Vascular Diseases | 3647 (4.4) | 3225 (4.4) | 0.766 |
| **Acute organ dysfunction count, median (IQR)** | 1 (1-2) | 1 (1-2) | <0.001 |
| **Acute organ dysfunctions, n (%)** |  |  |  |
| Renal | 51220 (62.1) | 44147 (60.1) | <0.001 |
| Elevated lactate | 36009 (43.6) | 32496 (44.2) | 0.021 |
| Hematologic | 8687 (10.5) | 11814 (16.1) | <0.001 |
| Hepatic | 10460 (12.7) | 9732 (13.2) | 0.001 |
| Circulatory (Shock) | 5863 (7.1) | 5964 (8.1) | <0.001 |
| Respiratory | 3605 (4.4) | 3475 (4.7) | 0.001 |
| **ICU admission, n (%)** | 23583 (28.6) | 22478 (30.6) | <0.001 |
| **Hospital LOS, median (IQR)** | 6 (4-10) | 6 (4-10) | <0.001 |
| **Discharge blood parameter, median (IQR)** |  |  |  |
| WBC (10^3^/µL) | 8.3 (6.20-10.70) | 8.48 (6.13-11.20) | <0.001 |
| Hemoglobin (g/dL) | 10.9 (9.40-12.40) | 10.5 (9.10-12.10) | <0.001 |
| Platelet count (10^3^/µL) | 218 (157-293) | 211 (144-290) | <0.001 |
| ANC (10^3^/µL) | 5.61 (3.90-7.80) | 5.74 (3.58-8.40) | 0.166 |
| ALC (10^3^/µL) | 1.32 (0.92-1.86) | 1.3 (0.86-1.80) | <0.001 |
| NLR | 4.06 (2.58-6.52) | 4.54 (2.69-7.79) | <0.001 |
| PLR | 161 (110-237) | 161 (107-242) | 0.826 |
| SII | 891 (493-1562) | 708 (301-1429) | <0.001 |
| **Base model inclusion criteria:** presence of all 8 CBCD parameters, hospitalizations were excluded if ≥1 CBCD parameters missing  P-value calculated comparing those included v. excluded from base model | | | |

**Supplemental Figure 2. Calibration plots for Base model (left) and Full model (right) for observed and predicted risk by outcome.**

| **Base Model** | **Full Model** |
| --- | --- |
| **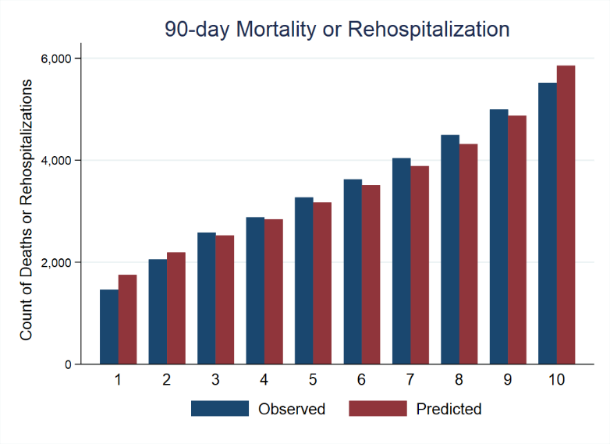** | **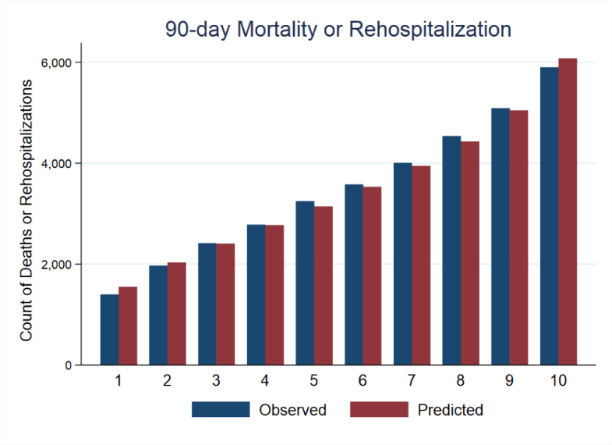** |
| **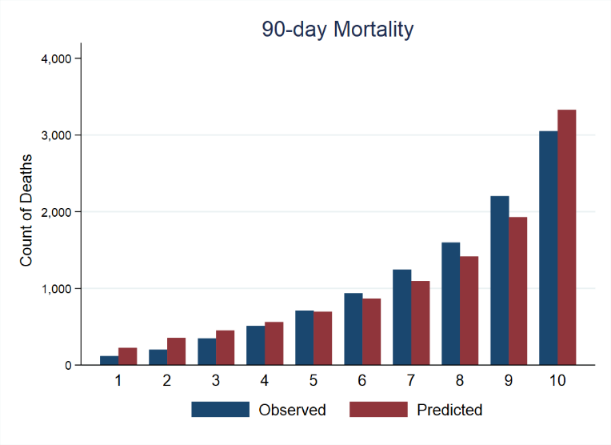** | **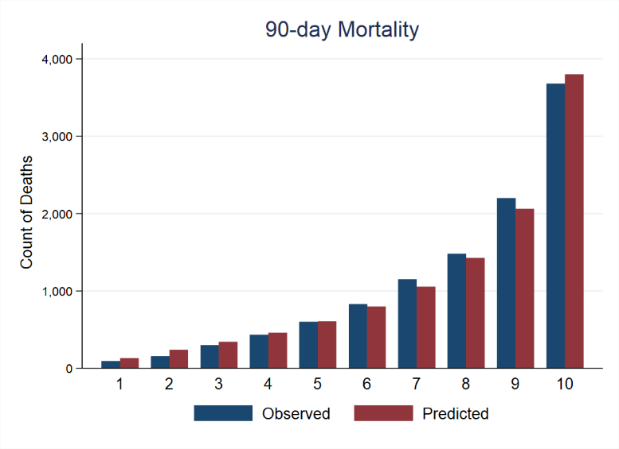** |
| **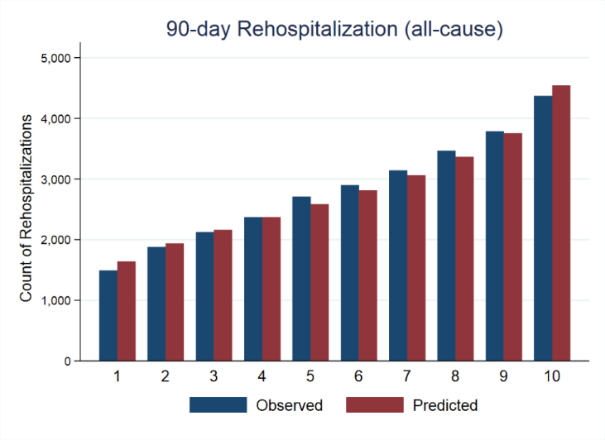** | **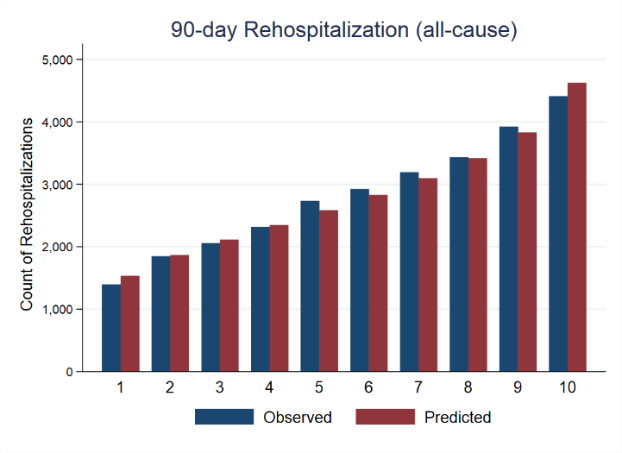** |
| **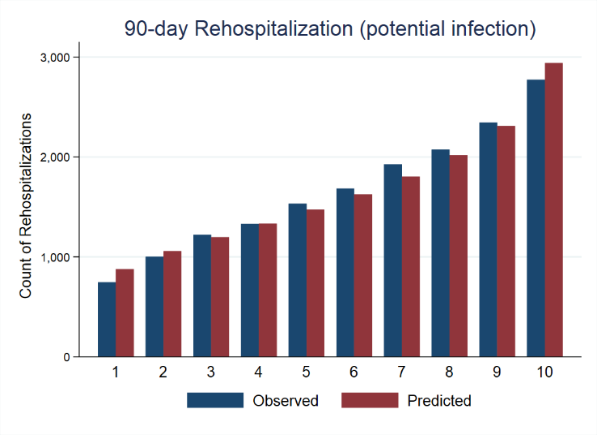** | **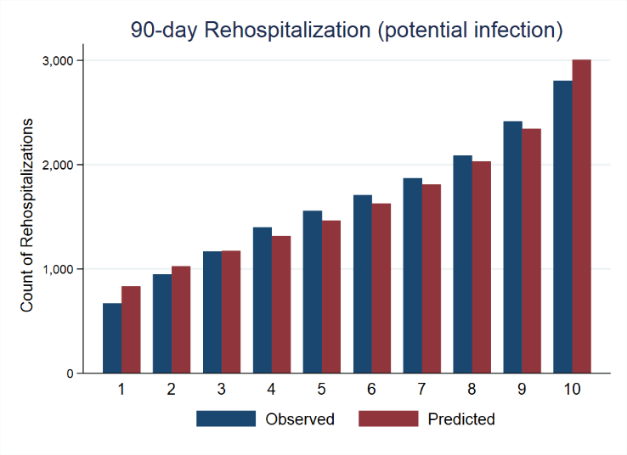** |
| **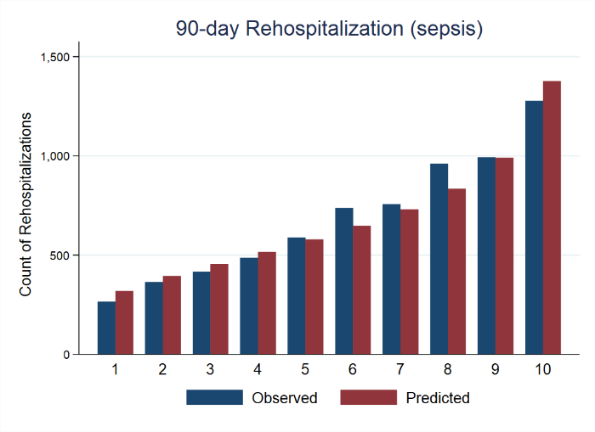** | **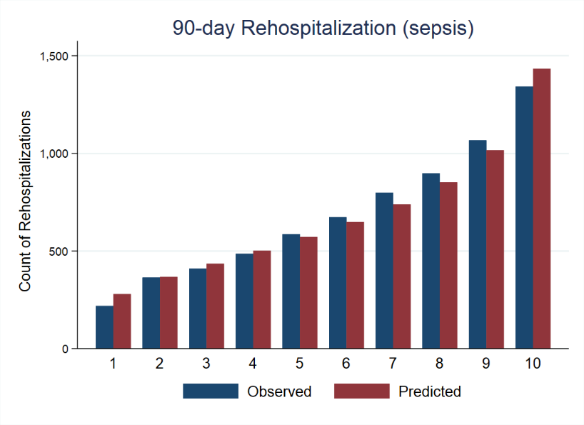** |

| **Supplemental Table 9: Observed vs predicted mortality or rehospitalization using 10 equally-size bins defined by decile of predicted risk using the Base model** | | | | | |
| --- | --- | --- | --- | --- | --- |
| **Risk Decile** | **Predicted risk (lowest, highest)** | **Hospitalizations, N** | **Observed Death or Rehospitalization, N (%)** | **Predicted Death or Rehospitalization, N (%)** | **Difference*, N (%)** |
| 1 | (0.127 – 0.243) | 8,251 | 1461 (17.71) | 1753 (21.25) | -292 (-3.54) |
| 2 | (0.243 – 0.287) | 8,251 | 2056 (24.92) | 2192 (26.56) | -136 (-1.64) |
| 3 | (0.287 – 0.325) | 8,251 | 2584 (31.32) | 2525 (30.6) | 59 (0.72) |
| 4 | (0.325 – 0.364) | 8,251 | 2882 (34.93) | 2844 (34.47) | 38 (0.46) |
| 5 | (0.364 – 0.405) | 8,251 | 3270 (39.63) | 3171 (38.44) | 99 (1.19) |
| 6 | (0.405 – 0.448) | 8,251 | 3626 (43.95) | 3511 (42.55) | 115 (1.4) |
| 7 | (0.448 – 0.495) | 8,251 | 4039 (48.95) | 3886 (47.09) | 153 (1.86) |
| 8 | (0.495 – 0.553) | 8,251 | 4494 (54.47) | 4317 (52.32) | 177 (2.15) |
| 9 | (0.553 – 0.633) | 8,251 | 4995 (60.54) | 4873 (59.06) | 122 (1.48) |
| 10 | (0.633 – 0.985) | 8,251 | 5519 (66.89) | 5854 (70.95) | -335 (-4.06) |
| 1-10 | (0.127 – 0.985) | 82,510 | 34926 (0.42) | 34926 (0.42) | 0 (0.00) |
| *Differences reflect the observed minus predicted mortality or rehospitalization. Negative values indicate that the model over-predicted mortality or rehospitalization at 90-days, while positive value indicate that the model under-predicted mortality or rehospitalization at 90-days.  The expected (mean) absolute calibration error across risk decile was 1.85%, while the maximum calibration error (observed in the highest risk decile) was 4.06%. | | | | | |

| **Supplemental Table 10: Observed vs predicted mortality or rehospitalization using 10 equally-size bins defined by decile of predicted risk using the Full model** | | | | | |
| --- | --- | --- | --- | --- | --- |
| **Risk Decile** | **Predicted risk (lowest, highest)** | **Hospitalizations, N** | **Observed Death or Rehospitalization, N (%)** | **Predicted Death or Rehospitalization, N (%)** | **Difference*, N (%)** |
| 1 | (0.100 – 0.221) | 8,251 | 1401 (16.98) | 1550 (18.79) | -149 (-1.81) |
| 2 | (0.221 – 0.269) | 8,251 | 1969 (23.86) | 2031 (24.61) | -62 (-0.75) |
| 3 | (0.269 – 0.314) | 8,251 | 2415 (29.27) | 2406 (29.16) | 9 (0.11) |
| 4 | (0.314 – 0.358) | 8,251 | 2782 (33.72) | 2771 (33.58) | 11 (0.14) |
| 5 | (0.358 – 0.404) | 8,251 | 3246 (39.34) | 3141 (38.07) | 105 (1.27) |
| 6 | (0.404 – 0.452) | 8,251 | 3577 (43.35) | 3529 (42.77) | 48 (0.58) |
| 7 | (0.452 – 0.506) | 8,251 | 4006 (48.55) | 3944 (47.8) | 62 (0.75) |
| 8 | (0.506 – 0.571) | 8,251 | 4538 (55) | 4431 (53.71) | 107 (1.29) |
| 9 | (0.571 – 0.658) | 8,251 | 5090 (61.69) | 5046 (61.15) | 44 (0.54) |
| 10 | (0.658 – 0.981) | 8,251 | 5902 (71.53) | 6078 (73.66) | -176 (-2.13) |
| 1-10 | (0.100 – 0.981) | 82,510 | 34926 (0.42) | 34927 (0.42) | -1 (0.00) |
| *Differences reflect the observed minus predicted mortality or rehospitalization. Negative values indicate that the model over-predicted mortality or rehospitalization at 90-days, while positive value indicate that the model under-predicted mortality or rehospitalization at 90-days.  The expected (mean) absolute calibration error across risk decile was 0.94%, while the maximum calibration error (observed in the highest risk decile) was 2.13%. | | | | | |

| **Supplemental Table 11.**  Sub-group analysis for area under the receiver operator characteristics (AUROC) for 90-day mortality or rehospitalization based on baseline comorbid diseases | | | | |
| --- | --- | --- | --- | --- |
| **Model**  AUROC (∆AUC) | **Overall cohort**  **N=82510** | **Excluding patients with cancer**  **N=61373** | **Excluding patients with baseline anemia**  **N=66513** | **Excluding patients with liver disease**  **N=67298** |
| **Base Model** | 0.6756 | 0.6706 | 0.6702 | 0.6753 |
| **Base + Hgb** | 0.6840 **(+0.0084)** | 0.6795 **(+0.0089)** | 0.6797 **(+0.0095)** | 0.6833 **(+0.0080)** |
| **Full Model** | 0.6929 **(+0.0173)** | 0.6871 **(+0.0165)** | 0.6880 **(+0.0178)** | 0.6910 **(+0.0157)** |
| **Clinical (Base) model:** logistic regression predicting 90-day outcome based on clinical characteristics including age, sex, individual comorbid conditions, acute organ dysfunction count, ICU use, and length of hospitalization.  **Full Model:** Base model + all 8 CBCD parameters  **Excluded Elixhauser comorbidities:**  **Cancer:** Lymphoma, metastatic cancer, solid tumor without metastasis  **Baseline anemia:** Blood loss anemia, deficiency anemia **Liver disease:** Liver disease  All models compared to base model using the Likelihood-ratio test: -2*ln*(likelihood of base model / likelihood base + CBCD model), p-value < 0.001 for all tests.  ∆AUC, delta area under the curve = AUROC_Base+CBCD_ – AUROC_Base_. Hgb, hemoglobin. | | | | |

| **Supplemental Table 12.**  **Likelihood ratio chi-squares for models including CBCD parameters as compared to the Base model for predicting 90-day outcome** | | | | | |
| --- | --- | --- | --- | --- | --- |
| **Model** | **90-day mortality or rehospitalization** | **90-day mortality** | **90-day rehospitalization (all-cause)** | **90-day rehospitalization (potential infection)** | **90-day rehospitalization (sepsis)** |
| Full (+ all 8 CBCD) | 1898.9 | 3309.8 | 610.8 | 387.4 | 319.2 |
| + Hgb | 868.1 | 868.1 | 475.2 | 161.9 | 181.5 |
| + NLR | 758.9 | 1830.7 | 30.9 | 28.9 | 15.3 |
| + SII | 464.3 | 886.7 | 29.8 | 42.9 | 23.6 |
| + ANC | 419.8 | 1201.5 | 29.5 | 81.7 | 47.4 |
| + ALC | 362.5 | 671.5 | 40.8 | 43.6 | 9.5 |
| + WBC | 289.7 | 797.6 | 36.4 | 115.6 | 52.8 |
| + Plt | 244.2 | 513.1 | 56.2 | 73.1 | 81.4 |
| + PLR | 185.9 | 257.3 | 46.7 | 33.7 | 20.9 |
| **Clinical (Base) model:** logistic regression predicting 90-day outcome based on clinical characteristics including age, sex, individual comorbid conditions, acute organ dysfunction count, ICU use, length of hospitalization.  **Full Model:** Base model + all 8 CBCD parameters  **Likelihood ratio test =** -2*ln*(likelihood of base model / likelihood base +CBCD model), p-value < 0.02 for all tests  WBC, total white blood cell count. Hgb, hemoglobin. Plt, platelet count. ANC, absolute neutrophil count. ALC, absolute lymphocyte count. NLR, neutrophil-to-lymphocyte ratio (NLR = ANC / ALC). PLR, platelet-to-lymphocyte ratio (PLR = Plt / ALC). SII, systemic immune-inflammation index (SII = ANC x PLR / ALC). | | | | | |
